# MANAGEMENT OF ACUTE SEVERE ULCERATIVE COLITIS: A CLINICAL UPDATE

**DOI:** 10.1590/0102-6720201600030017

**Published:** 2016

**Authors:** Carlos Walter SOBRADO, Lucas Faraco SOBRADO

**Affiliations:** Discipline of Coloproctology, Hospital das Clínicas, University of São Paulo Medical School, São Paulo, SP, Brazil

**Keywords:** Acute severe colitis, Proctocolitis, Toxic megacolon, Therapeutics.

## Abstract

**Introduction:**

Acute severe colitis is a potentially lethal medical emergency and, even today, its treatment remains a challenge for clinicians and surgeons. Intravenous corticoid therapy, which was introduced into the therapeutic arsenal in the 1950s, continues to be the first-line treatment and, for patients who are refractory to this, the rescue therapy may consist of clinical measures or emergency colectomy.

**Objective:**

To evaluate the indications for and results from drug rescue therapy (cyclosporine, infliximab and tacrolimus), and to suggest a practical guide for clinical approaches.

**Methods:**

The literature was reviewed using the Medline/PubMed, Cochrane library and SciELO databases, and additional information from institutional websites of interest, by cross-correlating the following keywords: acute severe colitis, fulminating colitis and treatment.

**Results:**

Treatments for acute severe colitis have avoided colectomy in 60-70% of the cases, provided that they have been started early on, with multidisciplinary follow-up. Despite the adverse effects of intravenous cyclosporine, this drug has been indicated in cases of greater severity with an imminent risk of colectomy, because of its fast action, short half-life and absence of increased risk of surgical complications. Therapy using infliximab has been reserved for less severe cases and those in which immunosuppressants are being or have been used (AZA/6-MP). Indication of biological agents has recently been favored because of their ease of therapeutic use, their good short and medium-term results, the possibility of maintenance therapy and also their action as a "bridge" for immunosuppressant action (AZA/6-MP). Colectomy has been reserved for cases in which there is still no response five to seven days after rescue therapy and in cases of complications (toxic megacolon, profuse hemorrhage and perforation).

**Conclusion:**

Patients with a good response to rescue therapy who do not undergo emergency operations should be considered for maintenance therapy using azathioprine. A surgical procedure is indicated for selected cases.

## RESUMO

**Racional:**

A colite aguda grave é emergência médica, potencialmente letal e o seu tratamento permanece ainda nos dias de hoje um desafio para o clínico e cirurgião. A corticoterapia intravenosa introduzida no arsenal terapêutico na década de 50 permanece como primeira linha de tratamento, e nos pacientes refratários a tal medida, a terapia de resgate pode ser com medidas clínicas ou colectomia de urgência.

**Objetivo:**

Avaliar os resultados da terapia de resgate medicamentosa (ciclosporina, infliximabe e tracolimus), suas indicações e resultados, e sugerir um guia prático para abordagem clínica.

**Métodos:**

Foi realizada revisão na literatura utilizando as bases Medline/Pubmed, Cochrane Library, Scielo, e informações adicionais em sites institucionais de interesse cruzando os descritores: colite aguda grave, colite fulminante e tratamento.

**Resultados:**

O tratamento da colite aguda grave tem evitado a colectomia em 60- 70% dos casos, desde que iniciado precocemente e com acompanhamento multidisciplinar. A ciclosporina intravenosa apesar de seus efeitos adversos, tem sido indicada naqueles casos mais graves com risco iminente de colectomia, pela sua rapidez de ação, meia-vida curta, e não aumentar os riscos de complicações cirúrgicas. A terapia com infliximabe tem sido reservada para os casos menos graves e naqueles em uso ou já expostos a imunossupressores (AZA/6-MP). A facilidade terapêutica, seus bons resultados a curto e médio prazo, a possibilidade de terapia de manutenção e também por agir como "ponte" para ação de imunossupressores (AZA/6-MP) tem recentemente favorecido a indicação de biológicos. A colectomia fica reservada para casos que não apresentaram resposta a terapia de resgate após cinco a sete dias de tratamento e nas complicações (megacólon tóxico, hemorragia profusa e perfuração).

**Conclusõe:**

s: Os pacientes com boa resposta à terapia de resgate e não submetidos à operações de urgência, deverão ser considerados para terapia de manutenção com azatioprina, sendo procedimento cirúrgico indicado para casos selecionados.

## INTRODUCTION

Ulcerative rectocolitis is characterized by a chronic inflammatory process of the colon and rectum. Despite the wide range of medications that is available to treat it, 15-20% of the patients will require hospitalization because of acute severe colitis^6^ ^,^ ^7^. It was described by Truelove e Witts, who used the following criteria to define it: diarrhea (>6 times a day), anal bleeding, fever (>37.8° C), tachycardia (HR>90 bpm), anemia (Hb<10.5 g/dl) and elevation of the erythrocyte sedimentation rate (ESR>30 mm)^28^. The term fulminating or toxic colitis is sometimes used as a synonym for acute severe colitis, but this should a priori be reserved for situations that are more severe and critical, in which significant septic conditions are observed, with more than 10 episodes of diarrhea/day and severe anemia requiring blood transfusions and imminent surgical treatment. The guidelines of the American College of Gastroenterology (Clinical Practice Guidelines, 2010) defined fulminating colitis as situations of diarrhea (>10 evacuations per day) in association with continuous rectal bleeding, systemic signs of toxicity (fever, tachycardia and hypotension), anemia that requires transfusion and abdominal pain with distension^14^. 

Toxic megacolon refers to states of acute abdominal pain associated with abdominal distension, such that simple abdominal radiographs show a dilated colon of >6 cm. This condition may be present in states of acute severe or fulminating colitis and generally requires emergency surgical treatment. It was first described in individuals with ulcerative rectocolitis in 1950 and may occur in approximately 15% of these patients. It is more frequent in cases of extensive colitis (macroscopic disease proximal to the splenic angle)^3^. 

This study had the aim of evaluating the indications for and results from drug rescue therapy (cyclosporine, infliximab and tacrolimus), and to suggest a practical guide for clinical approaches.

## METHODS

The literature was reviewed using the Medline/PubMed, Cochrane library and SciELO databases, and additional information from institutional websites of interest, by cross-correlating the following headings: acute severe colitis, fulminating colitis and treatment.

## RESULTS

### Etiology and physiopathology

Acute severe colitis is frequently associated with intestinal inflammatory disease. However, it may have various causes, such as infection, ischemia and intestinal pseudo-obstruction (Ogilvie). If a diagnosis of severe colitis is suspected, it is very important to rule out infectious causes due to cytomegalovirus, *Shigella, Salmonella, Entamoeba,* enterohemorrhagic *E. coli* and *Clostridium difficile.* In such cases, use of narcotics, non-steroidal anti-inflammatory drugs and antidiarrheal agents should be avoided, given that these may trigger toxic megacolon. Contrast enema and/or colonoscopy should also be avoided. Rectosigmoidoscopy with biopsy, with or without minimal insufflation, can be performed with the aim of ruling out cytomegalovirus. The latter infection is more common among patients with long exposure to corticoids and immunosuppressants.

The physiopathological mechanism that leads to colon distension remains somewhat unclear, but there is much evidence showing that the infectious process in the presence of colon ulceration leads to relaxation of the smooth muscle with absence of contraction and inhibition of the gastrocolic reflex, in response to the action of inhibitors of nitric oxide, vasoactive intestinal polypeptide (VIP) and substance P, with consequent dilatation^25^ ^-^ ^27^. Colon dilatation and the severity of the disease are factors that characterize toxic megacolon. In acute colitis, the inflammation is limited to the mucosal and submucosal layers, while in toxic megacolon the inflammatory process goes beyond the muscle layer, thus causing dilatation in the colon and possibly perforating the intestinal wall.

### Diagnosis

The diagnosis is suspected from the history and physical examination and is confirmed through laboratory tests (hemogram, platelets, urea, creatinine, sodium, potassium, albumin, ESR, C-reactive protein (CRP) and liver function tests) and through simple abdominal radiography, which may confirm the presence of toxic megacolon from the partial dilatation of the colon (right or transverse colon) or its total dilatation.

Laboratory tests are important in evaluating the inflammatory activity and also the degree of toxemia. In acute severe colitis or fulminating colitis, elevated CRP, thrombocytosis and hypoalbuminemia (<3.5 g/dl) can be found. CRP has been greatly used as a predictive factor regarding the therapeutic response to corticoid therapy, and also regarding the need for colectomy. Assays on cholesterol and serum magnesium should always be requested, since in the absence of any response to corticoids, it may be necessary to use cyclosporine. Patients with hypocholesterolemia (<120 mg/dl) and hypomagnesemia (<1.5 mg/dl) are at higher risk of presenting convulsive crises. Cyclosporine increases the depuration of magnesium, and this may lead to symptomatic hypomagnesemia, and especially to neurotoxicity and cardiac arrhythmias. It is therefore recommended that the serum magnesium levels should be controlled, particularly in the presence of neurological signs and symptoms. Among women of fertile age, it is always recommended that a pregnancy test should be undergone, because of the toxicity of the medication.

Feces examination for the presence of toxins A and B for *C*. *difficile* and rectosigmoidoscopy with biopsy to investigate cytomegalovirus are always indicated. If toxins for *C. difficile* are identified, oral treatment with metronidazole or vancomycin is started.

### Treatment

The treatment has the objectives of inducing clinical remission without corticosteroids, diminution of morbidity and mortality and improvement of quality of life. To attain these objectives, a multiprofessional approach with a proactive attitude (coloproctologist, gastroenterologist, nutrologist, psychologist and nurse) and adequate medication are important.

**FIGURE 1:**
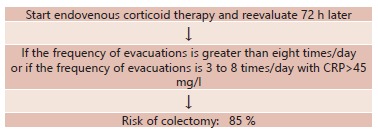
Response to corticoid therapy and risk of emergency colectomy^29^

Individuals with acute severe colitis need to be admitted to an intensive care unit and placed under a fasting regimen. Support measures need to be started (hydration, correction of hydroelectrolyte disorders and anemia), with sample collection for culturing (secretions, urine, feces and blood) and periodic reevaluations. Although antibiotic use is not routinely indicated for these patients, a wide-spectrum antibiotic regimen would always be used (ceftriaxone 2 g/day + metronidazole 500 mg every 8 h + ampicillin 1 g every 8 h; or ciprofloxacin 400 mg every 12 h + metronidazole 500 mg every 8 h + ampicillin 1 g IV every 8 h). This regimen can be modified according to the clinical evolution or the results from new cultures. Prevention of thromboembolic phenomena is of great importance, because it is known that venous and arterial vaso-occlusive events are important causes of morbidity and mortality among patients with intestinal inflammatory disease. In this regard the risk of intestinal bleeding must always be assessed. Prolonged immobilization needs to be avoided and use of central venous catheters should be minimized so as to avoid use of peripherally inserted central catheters (PICC) because of the high risk of thrombosis. Use of oral contraceptives and tobacco are suspended, vitamin replacement (B6, B12 and folic acid) is instituted and use of elastic stockings is started. Other thromboprophylactic measures such as prophylaxis with low molecular weight heparin (administered subcutaneously) are indicated for bedridden hospitalized patients. In situations of severe ulcerative colitis or fulminating colitis, parenteral corticoid therapy is the preferred treatment. Ever since the classical study published in 1955 by Truelove e Witts, which highlighted the benefits of steroids for treating states of acute ulcerative rectocolitis, these drugs have been greatly used for controlling active disease^28^. Before corticoid therapy was introduced, the mortality rate among individuals with severe ulcerative rectocolitis was 25%; nowadays it is 5-7%^12^. The daily dose of hydrocortisone is 300 mg, with methylprednisolone at a dose of 60 mg in the form of continuous infusion or as fractional doses. Approximately 30% of the patients will not present any improvement through endovenous steroids after 3-5 days and will be considered to be refractory to corticoid therapy^20^. Turner et al. (2007) showed through a meta-analysis evaluating the response to corticoid therapy among individuals with severe ulcerative rectocolitis that 67% presented a good clinical response^30^. Travis et al. sought to identify factors that might predict the response to corticoid therapy and, through evaluating 49 patients, concluded that the risk of requiring emergency colectomy was 85%, among patients who in assessments 72 h after the treatment continued to present more than eight evacuations/day, or three to eight evacuations/day in association with CRP > 45 mg/l ^29^ (Figure 1).

Other indexes using combinations of clinical data (frequency of evacuations, fecal consistency, anal bleeding, abdominal pain and need for transfusion), laboratory results (Hb, Ht, CRP, albumin and ESR) and radiological findings (abdominal x-ray showing dilatation of the colon) have been described as predictors of colectomy^1^. Lennard-Jones et al. observed that there was a need for colectomy in 55% of their patients, given that 48 h after administration of intravenous corticoids, they still presented more than 12 evacuations per day. They also reported that presence on simple abdominal radiography of colon dilatation greater than 5.5 cm or presence of mucosal islands and severe edema of the colon wall was associated with a need for colectomy in 75% of the cases^16^. Dinesen et al. reported from a retrospective study on 750 patients that the colectomy rate was associated with the severity of inflammatory activity at the time of hospital admission and with the number of hospitalizations. They concluded that the greater the number of clinical criteria that were associated with diarrhea with blood (>6 episodes/day), the higher the chance of requiring colectomy would be^5^ (Table 1).

**TABLE 1:**
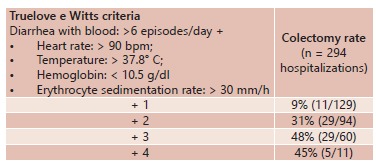
Correlation of colectomy with the severity of the clinical condition at the time of hospital admission and with the number of clinical criteria^1^

If the medical team concludes 48-72 h after administration of endovenous corticoid therapy that there has not been any response, rescue therapy (cyclosporine, infliximab or tacrolimus) should be started. Emergency surgical treatment is indicated in cases of clinical worsening or complications (profuse bleeding, perforation or toxic megacolon)^19^. 

Cyclosporine is a lipophilic peptide that inhibits calcineurin. It has been used since 1980 as an immunosuppressant for patients undergoing transplantation of solid organs. It started to be used for individuals with severe refractory ulcerative rectocolitis at the beginning of the 1990s. Lichtiger et al. conducted a placebo-controlled randomized study using cyclosporine (4 mg/kg/day) among patients with acute severe ulcerative rectocolitis that was refractory to corticosteroids. They found that 9/11 (81.8%) of the patients in the immunosuppressant group showed a clinical response, while none of the patients in the placebo group showed any response^17^. Their study was terminated early because of the poor initial results: five patients in the placebo group who migrated to the cyclosporine group also presented a good response. Other published studies with small samples and without controls have shown clinical response rates of the order of 64-86% ^24^. Cyclosporine has also been indicated in cases of Crohn's disease that is refractory to corticosteroids and in cases of its fistulizing pattern. The dose usually recommended is 2-4 mg/kg/day, administered through continuous endovenous infusion for 10-14 days and then becoming administered orally. Despite the good results obtained through cyclosporine, it has not been widely used in daily clinical practice, for the following reasons: high cost, severe adverse effects (convulsive crises, nephrotoxicity, cardiac arrhythmias, tremors, arterial hypertension, hyperkalemia, nausea and vomiting), opportunistic infections (*Pneumocystis carinii*), interaction with other drugs and the need for frequent monitoring. A study conducted in Mount Sinai Hospital, in New York, found serious infection in 5% of the cases and mortality in 1.8% ^26^. Attempts have been made to implement certain measures in order to reduce the risks of serious adverse effects, such as: diminishing the dose (2 mg/kg/day), avoiding hypocholesterolemia (by maintaining total cholesterol >150 mg/dl), avoiding hypomagnesemia, instituting prophylaxis against *P. carinii* (sulfamethoxazole-trimethoprim) and maintaining the cyclosporine level in the blood at between 200 and 400 ng/ml. Another important measure is to select the patients who are candidates for receiving cyclosporine: this should be avoided among patients with severe hypertension, kidney failure, active infection and signs and symptoms of neurological disorders, and among elderly patients with multiple comorbidities. According to some studies, endovenous cyclosporine avoids colectomy in 70-85% of these patients, with better results among those who make use of maintenance therapy consisting of AZA/6-MP^18^ ^,^ ^31^. Therefore, cyclosporine should not be indicated as long-term maintenance therapy but should serve as a bridge until full action by thiopurines or biological agents is attained. Cohen et al. showed that the likelihood of having avoided colectomy among individuals with acute severe colitis after five years of follow-up was greater among those who had received cyclosporine in association with AZA/6-MP (66%) than among those who only received cyclosporine. This suggested that there was some benefit from combining cyclosporine and an immunomodulatory^4^. Regarding the size of the appropriate dose for endovenous cyclosporine (2 or 4 mg/kg), a randomized double-blind study conducted by a Belgian group did not show any clinical benefits from a larger dose and showed that 85% of the patients in both groups had a favorable clinical response. Van Assche et al. concluded that 2 mg/kg should be the dose indicated. But that further studies using 1 mg/kg were needed in order to assess whether the efficacy is maintained ^31^. Use of endovenous cyclosporine should be maintained for 7-10 days, but the clinical improvement (diminution of diarrhea and bleeding) and the improvement in laboratory parameters generally occur between the third and fifth day. Thereafter, the medication should become orally administered (6-8 mg/kg/day), delivered every 12 h. 

Infliximab has also been used as rescue therapy among individuals with severe ulcerative rectocolitis that is refractory to endovenous corticoid therapy, Its use should be avoided among patients with tuberculosis (even if this is in its latent form), acute infections, seropositivity for hepatitis B and grades III and IV functional cardiac insufficiency. In a clinical study conducted by Sands et al. among patients with severe ulcerative rectocolitis that was refractory to steroids, who received infliximab, 50% showed a response whereas there was no response in the placebo group^21^. A randomized controlled multicenter study conducted in Scandinavia on 45 patients with severe ulcerative rectocolitis, in which patients with very severe disease that necessitated urgent colectomy, showed that three months after the treatment, the colectomy rate was lower in the infliximab group (29%; 7/24), which received a single dose of infliximab, vs. 67% (14/21) in the placebo group^11^. Three years later, these same 45 patients were reevaluated and the colectomy rates were found to be 50% in the infliximab group vs. 76% in the placebo group (p<0.05)^10^. In a retrospective Italian study on 85 patients with severe colitis, it was concluded that the patients who received two or more infusions of infliximab presented a better response with a lower colectomy rate (3/57) than did the group that received only one infusion (9/26; p=0.001)^13^.

A European multicenter study published in 2012 compared the treatment results from 115 individuals with severe ulcerative rectocolitis who were refractory to parenteral steroids, between a group that used cyclosporine (2 mg/kg/day) for three months and a group that used infliximab (5 mg/kg/dose) in three infusions (0, 2 and 6 weeks). The initial responders were maintained using azathioprine starting on the seventh day of treatment. In the cyclosporine group, 60% did not present any clinical response, while in the infliximab group failure occurred in 54% (p>0.05)^15^. It is important to emphasize that, just as was seen with cyclosporine, the infliximab group also showed a not inconsiderable percentage of side effects such as respiratory infections, tuberculosis and lymphoma. This topic is difficult to assess because the great majority of these patients had also previously used high doses of corticosteroids and some had used azathioprine, which may have been the cause of the complications.

The choice between cyclosporine and infliximab in cases that are refractory to endovenous corticoid therapy is difficult. It will depend on the patient's clinical conditions and the experience of the medical-surgical team with these medications. One of the hypothetical disadvantages of using infliximab is its longer half-life, which may be a risk factor for surgical complications, if a need for an emergency operation were to arise. For patients with very severe ulcerative rectocolitis, it seems that cyclosporine is preferable because of its faster action, short half-life and good clinical response in 70-80% of the patients, particularly for those who are virgin to treatment with AZA/6-MP and those with a high likelihood of surgery. On the other hand, infliximab is the preferred medication for patients with less severe conditions or those with indeterminate colitis who have previously used and have presented failure or intolerance regarding AZA/6-MP. Some studies have been conducted with the aim of determining the factors that might, when present, predict the need for colectomy among patients who are using infliximab. It was concluded that elevation of CRP (>20 mg/l), concomitant use of corticoids, ulcerative rectocolitis of less than three years of duration, Mayo score >10 points, presence of anti-infliximab antibodies and serum levels of the drug undetectable after the first infusion were associated with high rates of colectomy^22^.

Another drug that has been used in cases that are refractory to corticoid therapy is tacrolimus, which is a calcineurin inhibitor with a mechanism of action similar to that of cyclosporine. Some studies have shown results similar to those from cyclosporine both through endovenous administration (0.01 to 0.02 mg/kg) and through oral administration (0.1 to 0.2 mg/kg) ^8^. Fellermann et al, at the University of Stuttgart, used tacrolimus as rescue therapy for 38 patients and found that 18 of them showed a good clinical response after two weeks, such that 13 of them entered clinical remission within 30 days. Colectomy was necessary in 34% of the cases, and in 8% (3/38) within the first month. They concluded that tacrolimus was equally efficient and safe, whether taken orally or parenterally^8^. Baumgart et al. conducted a study on a small number of patients with severe refractory ulcerative rectocolitis and reported that after 44 months of follow-up, colectomy had been avoided in 57%^2^.

Surgical treatment is reserved for cases of clinical worsening or absence of improvement 5-7 days after the drug treatment, and for case of complications (profuse hemorrhage with hemodynamic instability, perforation or toxic megacolon). The main cause of surgical indications is clinical untreatability. Subtotal colectomy with burial of the rectum at the level of the peritoneal reflection and with ileostomy is the preferred technique, given that this avoids dissection of the rectum (which is generally very inflamed) and prevents iatrogenic lesions of the pelvic nerve plexus (which would produce the risk of sexual and urinary dysfunction). Anastomoses should be avoided in emergency operations, especially among immunosuppressed patients with acute conditions who are refractory to intensive treatment, and among those who were using prednisone at doses greater than 20 mg/day over the six-week period preceding the operation. In these cases, colectomy not only saves the patient but also diminishes the risk of colorectal cancer. Patients are reevaluated 3-6 months later, with a view to performing proctectomy and a J-shaped ileal pouch. Although ileoanal anastomosis with a reservoir is considered to be the treatment that "cures" ulcerative rectocolitis and improves quality of life, it is associated with high morbidity, such as: 4-8 evacuations/24 h, fecal escape, nocturnal incontinence, reduction of female fertility, bursitis and irritable bowel syndrome^9^.

### Toxic megacolon 

Toxic megacolon is a complication of severe ulcerative rectocolitis that is characterized by acute non-obstructive partial or total dilatation of the colon (colon with diameter >5.5 cm), associated with signs of toxemia (fever, tachycardia, pain, abdominal distension, mental confusion, anemia and leukocytosis). It may also be a consequence of Crohn's colitis, infectious colitis (*C. difficile*, cytomegalovirus and salmonella) or ischemic colitis. Its incidence ranges from 5-17% among hospitalized patients and its risk factors comprise use of narcotics, opiates, anti-diarrheal agents, anti-cholinergic agents, anti-inflammatory agents, hypokalemia, hypomagnesemia and opaque enema or colonoscopy performed at the time of acute transformation^9^. From a physiopathological point of view, the toxemic state results from diminished motility, colon dilatation and fecal ectasia with bacterial translocation. It is detected through clinical suspicion and radiological examination of the abdomen confirms the diagnosis, since it reveals loss of haustration, edema of the wall and colon dilatation. Tomography of the abdomen is very useful because it may reveal abdominal complications (associated tumors, free fluid in the cavity and pneumoperitoneum) that are difficult to confirm in immunosuppressed patients who are using high doses of corticoids. Toxic megacolon is a potentially lethal condition if not diagnosed and treated efficiently, early on. The presence of toxic megacolon is not an absolute indication for an emergency operation: it depends on the coloproctologist's assessment and in many situations can be managed as described earlier for severe colitis.

The treatment consists of support measures (hydration, correction of hydroelectrolytic disorders, correction of anemia and nutritional support), fasting, nasogastric probes, broad-spectrum antibiotic therapy and endovenous corticoid therapy. Some authors have advocated use of intravenous cyclosporine and oxygen therapy, but there is still no robust scientific evidence to support these treatments^9^. Endovenous proton pump inhibitors and prophylaxis against thrombosis (subcutaneous heparin and elastic stockings) are necessary measures. In patients with toxic megacolon resulting from *C. difficile*, the antibiotic currently in use should be suspended. Endovenous metronidazole should be started (500 g every 8 h) and vancomycin should be applied via a nasogastric probe or orally.

**FIGURE 2:**
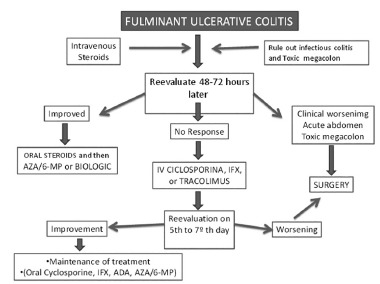
Proposed algorithm for treating acute severe colitis and fulminant ulcerative colitis.

If no improvement occurs within a 24-48 h period, or signs of intestinal perforation occur, colectomy is indicated. If an operation is performed early on, without intestinal perforation, the mortality rate is of the order of 1-8%, but in cases of perforation of the colon with peritonitis, it reaches 40-50% ^23^. Total colectomy with terminal ileostomy and burial of the rectum at the level of the peritoneal reflection, in association with drainage of the cavity in cases of peritoneal contamination is the preferred treatment in emergency cases. After clinical improvement, and with confirmation of ulcerative rectocolitis through histological examination of the surgical specimen, the definitive treatment can be indicated: ileal pouch-anal anastomosis with a J-shaped reservoir, Figure 2).

IFX=infliximab; ADA=adalimumab; AZA=azathioprine; 6-MP=6 mercaptopurine

## CONCLUSIONS

Patients with acute severe colitis with a good response to rescue therapy who do not undergo emergency operations should be considered for maintenance therapy using azathioprine. A surgical procedure is indicated for selected cases.
